# Placental Oxidative Status throughout Normal Gestation in Women with Uncomplicated Pregnancies

**DOI:** 10.1155/2015/276095

**Published:** 2015-02-01

**Authors:** Jayasri Basu, Bolek Bendek, Enyonam Agamasu, Carolyn M. Salafia, Aruna Mishra, Nerys Benfield, Ronak Patel, Magdy Mikhail

**Affiliations:** Department of Obstetrics & Gynecology, Bronx Lebanon Hospital Center, 1650 Grand Concourse, Bronx, NY 10457, USA

## Abstract

The effects of gestational age on placental oxidative balance throughout gestation were investigated in women with uncomplicated pregnancies. Placental tissues were obtained from normal pregnant women who delivered at term or underwent elective pregnancy termination at 6 to 23 + 6 weeks of pregnancy. Placental tissues were analyzed for total antioxidant capacity (TAC) and lipid peroxide (malondialdehyde, MDA) levels using commercially available kits. Two hundred and one placental tissues were analyzed and the mean ± SD MDA (pmol/mg tissue) and TAC (*µ*mol Trolox equivalent/mg tissue) levels for first, second, and third trimester groups were 277.01 ± 204.66, 202.66 ± 185.05, and 176.97 ± 141.61, *P* < 0.004 and 498.62 ± 400.74, 454.90 ± 374.44, and 912.19 ± 586.21, *P* < 0.0001 by ANOVA, respectively. Our data reflects an increased oxidative stress in the placenta in the early phase of normal pregnancy. As pregnancy progressed, placental antioxidant protective mechanisms increased and lipid peroxidation markers decreased resulting in diminution in oxidative stress. Our findings provide a biochemical support to the concept of a hypoxic environment in early pregnancy. A decrease in placental oxidative stress in the second and third trimesters appears to be a physiological phenomenon of normal pregnancy. Deviations from this physiological phenomenon may result in placental-mediated disorders.

## 1. Introduction

During the first trimester of pregnancy, the conceptus develops in a low oxygen environment that favors organogenesis in the embryo and angiogenesis in the placenta [1]. This low oxygen environment is created when maternal arterial blood is prevented from entering the intervillous space of the placenta by plugs of cytotrophoblast cells that invade the uterine spiral arteries. As pregnancy continues, higher concentrations of oxygen are required to support the rapid growth of the fetus and the placenta. At the end of first trimester, the maternal intraplacental circulation is fully established when cytotrophoblast cell plugs are dislodged, by an unknown mechanism and maternal blood flow to the intervillous space ensues. The normoxic environment thus created in the placenta is then maintained until term [1]. Perturbations in such an oxidative environment as pregnancy continues are suggested to play a role in the pathophysiology of pregnancy disorders such as preeclampsia, intrauterine growth restriction, and early pregnancy loss [1].

The altered hormonal status during pregnancy results in increased accumulation of maternal fat depots and hyperlipidemia [2]. Maternal plasma triglycerols and nonesterified fatty acids are reported to correlate with fetal lipid and fetal growth, suggesting that these molecules do traverse through the placenta [3]. The abundant presence of membrane phospholipids at sites where reactive oxygen species are formed makes them easily accessible endogenous targets for lipid peroxidation [4]. The free radical chemistry of lipid peroxidation is complex. Lipid peroxidation occurs as a chain reaction initiated by free radicals, which propagates itself and can result in the formation of many equivalents of lipid peroxides. MDA is one of the several low-molecular-weight end products formed from the decomposition of certain primary and secondary lipid peroxidation products. Nevertheless, MDA is not exclusively generated through lipid peroxidation. Oxidative modification of lipid can be induced* in vitro* or could occur* in vivo* during aging [5] and certain diseased conditions [6, 7]. The host of other lipid peroxidation products include: diene hydroperoxide, cyclic peroxides, bicyclic peroxides, or epoxy alcohol [7]. Of all these products of lipid peroxidation, MDA and 4-hydroxynonenal are naturally occurring biproducts. The determination of MDA is rather fairly simple and prompt. MDA readily participates with 2-thiobarbituric acid at a low pH and an elevated temperature. The reaction product is a 1 : 2 MDA-TBA adduct that is red in color, which can readily be determined colorimetrically. The ease of the assay has fostered MDA determination to be the most widely employed format for monitoring lipid peroxidation in a wide array of samples.

Efforts in understanding the increased oxidative stress during pathological states of pregnancy have been the focus of many studies in recent times. For this, investigators have measured lipid peroxides and antioxidant levels either in the blood or in the placenta of women, primarily with preeclampsia, and have compared the levels to that of normal women with uncomplicated pregnancies [8–12]. However, information regarding the change in placental oxidative stress throughout pregnancy is not well-studied. The objective of the present study was to investigate the effects of gestational age on the oxidative balance throughout gestation in women with uncomplicated pregnancies. In this study, the levels of placental malondialdehyde (MDA) were determined as a marker of lipid peroxidation and reactive oxygen species (ROS). Since a large number of antioxidants are present in our body in the form of several micro and macro molecules, as well as enzymes, and these molecules function synergistically in preventing oxidative stress, hence quantitative measurement of the total antioxidant capacity (TAC) of the placenta was simultaneously measured as a marker for antioxidant defenses.

## 2. Materials and Methods

The investigative protocol for this study was approved by the Institutional Review Board of the Bronx Lebanon Hospital Center. A total of 201 placental tissues were obtained from either normal pregnant women who delivered at term or from women who underwent elective pregnancy termination at 6 to 23 weeks and 6 days of pregnancy. Placental tissues from women with a history of hypertension or pregnancies that were complicated by diabetes, peripheral vascular disease, chronic renal disease, multifetal gestation, or major fetal anomalies were excluded from the study.

Placental samples were collected within 10 minutes of completion of the procedure. The locations of the placental tissues obtained following elective termination of pregnancies are unknown; however, placental tissues obtained in the third trimester were taken from one of the peripheral cotyledons. Each sample was washed thoroughly in saline to remove maternal blood and was then dissected in saline to identify chorionic villi without associated decidua. Villous samples were transported to the laboratory on ice and stored at −80°C until assay. The placental tissues were analyzed for TAC using TAC assay kit from Sigma Aldrich (St. Louis, MO, Catalog number CS0790). The principle of the assay is that, in the presence of hydrogen peroxide, metmyoglobin forms ferryl myoglobin radical which oxidizes ABTS (2,2′-azino-bis(3-ethylbenzthiazoline-6-sulfonic acid)) to produce a radical cation ABTS^·+^. ABTS is colorless but when ABTS^·+^ is produced a soluble green colored chromogen is formed that can be determined spectrophotometrically at 750 nm. Antioxidants suppress the production of the radical cation in a concentration dependent manner and the color intensity decreases proportionately. Trolox, a water soluble vitamin E analog, is used in the assay to serve as a standard or control antioxidant. The lipid peroxidation (MDA) assay kit used for the study was obtained from Abcam (Cambridge, MA, Catalog # ab118970). The principle of the MDA assay is that MDA present in the sample is reacted with thiobarbituric acid (TBA) to generate MDA-TBA adduct. The MDA-TBA adduct is then quantified spectrophotometrically. For each assay, tissue homogenate was prepared using the buffer supplied with each kit and the assays were carried out as per the manufacturers' instructions. A Tecan Infinite 200 Pro Microplate Reader was used for the assays. For MDA, the absorbance of the color was measured at 532 nm. MDA levels were expressed as pmol/mg tissue. For TAC, the absorbance was measured at 750 nm and the results were expressed as *μ*mol Trolox equivalent/mg tissue. The intra-assay and interassay variations for both MDA and TAC were between 2–5% and 5–7%, respectively.

## 3. Statistical Analysis

Statistical evaluation of the data was carried out using SPSS^R^ statistical package version 22 [13]. Descriptive statistics was performed. Since the data was not normally distributed, Kruskal-Wallis test was performed to compare the differences among three trimester groups followed by Mann-Whitney *U* test for intergroup comparisons. Spearman's bivariate correlation was used to determine correlation between the MDA, TAC and gestational age in days and partial correlation was performed to determine the correlation between MDA and TAC after controlling for gestational age in days. A value of *P* < 0.05 was considered statistically significant.

## 4. Results

In the study, a total of 201 placentas were evaluated for both MDA and TAC levels. Of these, 106 placental tissues were collected from women up to 13 weeks of gestation and were grouped as first trimester; 48 placental tissues were collected from women from 13^+^ to 23 weeks + 6 days of gestation and were grouped as second trimester; and 47 tissues were obtained from term normal placentas which were selected from deliveries of a newborn without maternal or fetal-neonatal pathologies and were grouped as third trimester.

**Figure 1:**
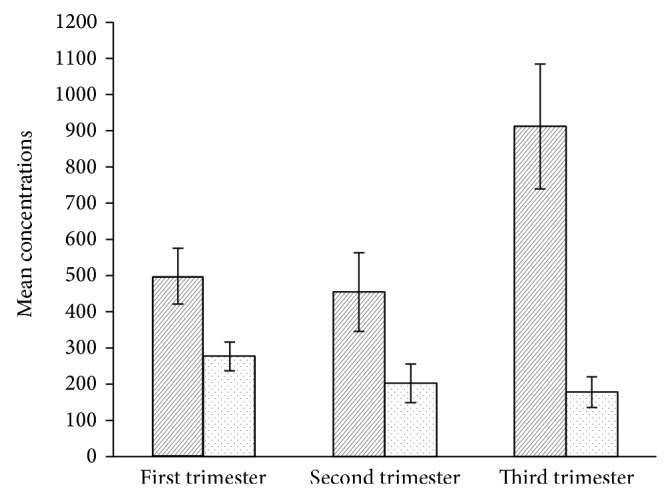
MDA: malondialdehyde; TAC: total antioxidant capacity. A box plot representating mean placental concentrations of TAC and MDA levels throughout gestation in normal pregnancy. The hatched bars represent mean placental TAC levels (*μ*mol Trolox equivalent/mg tissue); the dotted bars represent mean placental MDA levels (pmol/mg tissue); and the error bars denote 95% CI.

**Figure 2:**
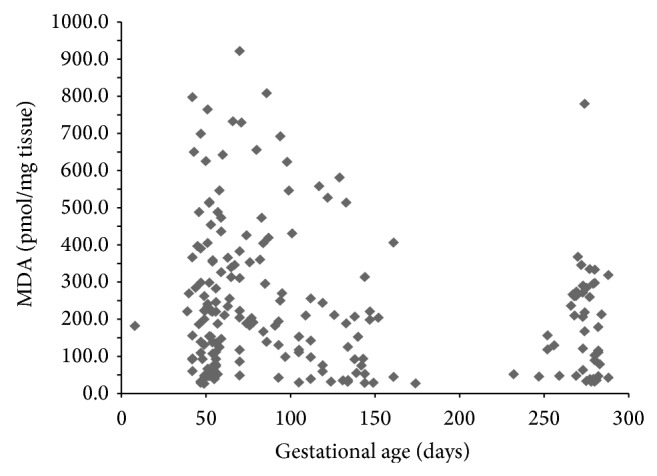
MDA: malondialdehyde. The scatter plot showing a downward trend in placental MDA levels with an increase in gestational age in days.

**Figure 3:**
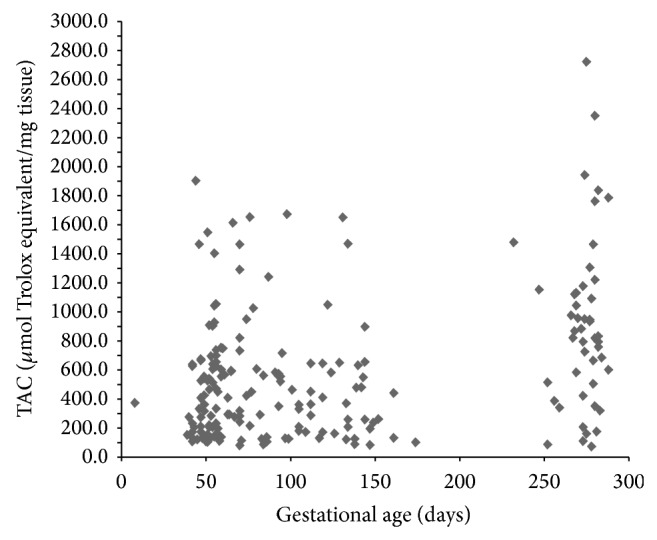
TAC: total antioxidant capacity. The scatter plot showing an upward trend in placental TAC levels with an increase in gestational age in days.

Placental oxidative status throughout normal gestation is presented in Table 1. Placental MDA levels were the highest in the first trimester of normal pregnancy. In the second and third trimesters, placental MDA levels were progressively lower. Placental TAC levels on the other hand were found to be significantly higher in the third trimesters. A bar plot and depicting the effects of gestational age on placental MDA and TAC levels by trimester is presented in Figure 1. The mean maternal age between the trimester groups as shown in Table 1 was not significantly different. Kruskal-Wallis test (Table 2) revealed significant differences in both placental MDA and TAC levels (*P* < 0.003, *P* < 0.0001, resp.), among the three trimester groups. Pairwise comparisons of MDA and TAC levels by trimester groups were performed using Mann-Whitney *U* test and the results are presented in Tables 3 and 4, respectively. While the placental MDA levels were noted to decrease with an increase in gestational age, the TAC levels on the other hand showed an increase. Two scatter plots depicting the MDA and TAC levels with gestational age in days are presented in Figures 2 and 3. A downward trend in MDA and an upward trend in TAC levels can be seen as pregnancy progressed. Spearman's bivariate correlation computed to determine the correlation between placental MDA and TAC levels with gestational age (GA, in days) showed a negative significant correlation between MDA and gestational age (*r* = −0.191, *P* < 0.007). TAC and gestational age were positively correlated and the correlation was significant (*r* = 0.262, *P* < 0.0001). The correlation between MDA and TAC levels was negative (*r* = −0.065, 0.362) (Table 5). Partial correlation after controlling for gestational age between TAC and MDA was not significant (*r* = 0.067, *P* = 0.345).

## 5. Discussion

The results of the present study demonstrate that placental MDA levels were the highest in the first trimester of normal pregnancy. Thereafter, the levels showed a gradual decline as pregnancy continued and there was a negative correlation between placental MDA levels and gestational age (Table 5). On the other hand placental TAC levels were found to be the lowest in the first and second trimester but the levels steadily increased as pregnancy progressed (Table 1 and Figure 1). Our data are in agreement with an* in vitro* study which demonstrated that the production of lipid peroxide peaked in early placental samples but, by the end of pregnancy, there was minimal lipid peroxide produced [14]. In another previously reported study, the levels of conjugated diene, a breakdown product of lipid peroxidation, in the serum was found to rise more than 45% in the second trimester over the first trimester values but by the third trimester the levels declined [15].

Since lipid peroxide levels of the placenta in our study were the highest in the first trimester while the TAC levels were the lowest, the findings provide a biochemical support of the concept of the existence of physiologic hypoxia in the placenta during the first trimester. Our findings are in agreement with other nonbiochemical studies that also reported placental hypoxia in the first trimester [1, 16–18]. Morphological studies have shown that placental hypoxia occurs in the first trimester when extravillous trophoblast plugs block the maternal spiral arteries and prevent the maternal blood from entering the intervillous space [16]. In normal pregnancies, Doppler studies have confirmed the absence of blood flow into the intervillous space prior to 10 weeks of gestation [17]. Additionally, direct comparison of oxygen tension between the placenta and its adjacent endometrial tissue also revealed that the partial pressure of oxygen in the placenta was significantly lower compared to that of the endometrium [18]. Investigators believe that such a hypoxic environment in the first trimester is essential for the regulation of trophoblast differentiation, embryogenesis, and/or placental development for a normal pregnancy outcome. The oxidative status of the placenta is not geographically homogenous. At high altitude, where the partial pressure of oxygen is low, placental hypoxic stress is induced that thwarts the high-altitude pregnancies more toward a preeclamptic phenotype [19].

For normal pregnancy to progress efficiently, the transition from a hypoxic to a normoxic environment is vitally important. During first trimester, antioxidant mechanisms are induced in the placenta to neutralize the excess accumulation of ROS and to trigger mechanisms that can remove or repair any damaged cells [20]. Our study shows that placental oxidative stress wanes off as normal pregnancy continues. By third trimester, a progressive increase in TAC levels was noted with a simultaneous decrease in MDA levels compared to the first trimester values (Tables 1, 3, and 4). Similar results of higher serum TAC levels in the third trimester of normal pregnancy over the first trimester values have also been previously reported [21]. Our results indicate that by the third trimester placental antioxidant protection mechanisms become efficient enough to counteract the oxidative challenge. Failure to establish such efficient antioxidant protection may contribute to the development of preeclampsia.

Low oxygen environment favors the generation of ROS which are characterized as molecules having one or more unpaired electrons [22]. The unpaired electron gives considerable reactivity to the free radicals and their nonradical intermediates. Recent* in vitro*, cell culture, and animal model studies have confirmed that ROS can activate a variety of transcription factors and protein kinases, influence the expression of a number of genes, are involved in signal transduction pathways, and can act as subcellular messengers for certain growth factors [22–26]. Placental adaptation in response to low oxygen tension occurs early in the pregnancy and during the process ROS act as key cell signaling molecules [24–26]. The increased levels of lipid peroxide in the first trimester of pregnancy in our study could reflect a period when excessive production of ROS might have taken place (Table 1). The physiological role of ROS during early pregnancy is suggested to influence a number of functions including remodeling of the spiral arteries, angiogenesis, and proliferation of the cytotrophoblast cells. Additionally, increased ROS in the placenta in the early phase of normal pregnancy has been shown to alter cell permeability and vascular biology, stimulate cellular and matrix remodeling, and protect the fetus from intrauterine infection [24–27]. The increased production of ROS in the first trimester of normal pregnancy could induce cytotrophoblast proliferation and the expression of key developmental genes involved in embryogenesis and placental development. This increase in cytotrophoblast proliferation in the first trimester is suggested by Douglas and Haddad [28] to be a natural protective mechanism to establish a mature placenta in advance of the period of rapid fetal growth.

Our study has limitations. The short questionnaire that was approved by our Institutional Review Board for the study included collecting information on maternal age, parity, gestational age as confirmed by ultrasound, and infection status. Smoking status of the patients, though was included in the questionnaire, was, however, not strictly monitored. Moreover, information on dietary and/or vitamin intake throughout the gestational period was not included. It is noteworthy, however, that the catchment area of the Bronx from where the pregnant women came from comprised primarily of African American (38%) and Hispanic (52%) descent. A previous study carried out on women from the same catchment area of the Bronx revealed that dietary and vitamin intakes were comparable between the pregnant and the nonpregnant groups (unpublished data).

Oxidative stress status in human placentas throughout normal pregnancy has not been well studied previously. Correlating the fetal blood oxidative status to the placenta and the placenta to the maternal systemic blood could ideally reflect the placental oxidative status and perhaps would be highly desirable. The focus of this study was in understanding the placental oxidative status throughout normal gestation. Comparative investigation of cord blood and maternal blood samples to identify any association can only be carried out in late stage of pregnancy and needs to be addressed in future studies. Our findings demonstrate that a decrease in oxidative stress with increase in gestational age may be a normal physiological phenomenon of normal pregnancy. Our data demonstrate the presence of a placental oxidative stress in the first trimester when the placental MDA levels are significantly high, with minimum protection offered by placental antioxidant system. As pregnancy progresses, the placental environment switches to a normoxic state as reflected by a gradual increase in the total antioxidant capacity of the placenta and a decrease in lipid peroxidation markers. By the third trimester, our results show that placental antioxidant mechanisms become more efficient to counteract the oxidative challenge. Deviation from this physiological balance whether caused by increased generation of ROS or decreased neutralization by antioxidants can cause placental damage and may contribute to abnormal pregnancy outcome.

## Figures and Tables

**Table 1:** Placental MDA and TAC levels throughout normal gestation.

Groups	*N*	MDA	TAC	Age
		(pmol/mg tissue)	(*μ*mol Trolox equivalent/mg tissue)	(yrs)
First trimester	106	277.01 ± 204.66	498.62 ± 400.74	26.77 ± 6.27
Second trimester	48	202.66 ± 185.05	454.90 ± 374.44	25.87 ± 6.50
Third trimester	47	176.97 ± 141.61	912.19 ± 586.21	26.83 ± 6.33

MDA: malondialdehyde; TAC: total antioxidant capacity. Data expressed as mean ± SD.

Placental MDA levels were highest in the first trimester and progressively declined thereafter, while TAC levels showed a significant increase beyond the second trimester of normal pregnancy.

**Table 2:** Comparison of placental MDA and TAC levels by trimester groups.

Groups	*N*	MDA	TAC
		Mean rank	Mean rank
First trimester	106	114.39	91.44
Second trimester	48	86.81	85.52
Third trimester	47	85.30	138.37

MDA: malondialdehyde; TAC: total antioxidant capacity. Kruskal-Wallis test results.

The critical values for MDA and TAC levels were 11.895 (*P* < 0.003) and 25.665 (*P* < 0.0001), respectively.

**Table 3:** Pairwise comparison of placental MDA levels.

	First trimester	Second trimester	Third trimester
	Test Score, *P* value	Test Score, *P* value	Test score, *P* value
First trimester	—	1885.0, 0.01	1731.0, 0.003
Second trimester		—	1106.0, 0.87
Third trimester			—

MDA: malondialdehyde; the nonparametric Mann-Whitney *U* test was applied.

Significant differences were noted between first and second trimester (*P* < 0.01) and first and third trimester (*P* < 0.003) groups. The difference in MDA levels between second and third trimester groups was not significant.

**Table 4:** Pairwise comparison of placental TAC levels.

	First trimester	Second trimester	Third trimester
	Test score, *P* value	Test score, *P* value	Test score, *P* value
First trimester	—	2394.0, 0.558	1327.5, 0.0001
Second trimester		—	535.0, 0.0001
Third trimester			—

TAC: total antioxidant capacity; the nonparametric Mann-Whitney *U* test was applied.

Significant differences were noted between first and third trimester (*P* < 0.0001) and second and third trimester (*P* < 0.0001) groups. The difference in TAC levels between first and second trimester groups was not significant.

**Table 5:** Correlation between MDA, TAC, and gestational age.

	MDA		TAC		GAD	
	Rho	*P*	Rho	*P*	Rho	*P*
MDA	1.000	—	−0.065	0.362	−0.191	0.007
TAC			1.000	—	0.262	0.0001
GAD					1.000	—

MDA: malondialdehyde; TAC: total antioxidant capacity; GAD: gestational age in days.

The nonparametric test Spearman's correlation was applied. Results show significant negative correlations between MDA and GAD and significant positive correlation between TAC and GAD. MDA and TAC levels were negatively correlated, but the correlation was not statistically significant.
